# Aging Veterans: Physical Health, Mental Well-Being, and Social Connections

**DOI:** 10.1093/geroni/igaf122.625

**Published:** 2025-12-31

**Authors:** Janet Wilmoth

## Abstract

Although the aging veteran population has received more scholarly attention in recent years, gerontologists continue to disentangle the confounding and sometimes countervailing influence of military service on later-life outcomes. This symposium provides insights into the physical health and mental well-being of aging veterans, while also offering insight into strategies for promoting social connections among rural veterans. Serier and colleagues, using a sample of Vietnam era veterans, find posttraumatic stress disorder (PTSD) is associated with hypertension and hypertension treatment among male veterans but not female veterans. London et al. estimate the joint influence of veteran status and PTSD on cardiovascular disease (CVD) risk with data from the National Wellbeing Survey. The results demonstrate increased CVD risk for veterans and those with PTSD, with the highest risk observed among veterans with PTSD. Marini and colleagues report findings from a primary data collection focused on how daily emotions are impacted by thoughts. They find between-person and within-person associations between thinking and talking about service experiences, rumination, and negative affect. Using Veteran Affairs healthcare system data, Guneet et al. find rural veterans are more likely to have a cognitive impairment diagnosis and are more likely to receive dementia medication, even prior to a pre-dementia or dementia diagnosis. Pless Kaiser and colleagues discuss a social group intervention designed to improve social connections among older rural Veterans with PTSD. They describe implementation of the program at two sites in Montana and North Carolina then offer suggestions for program dissemination.

